# Perceived classism and its relation with socioeconomic status, health, health behaviours and perceived inferiority: the Dutch Longitudinal Internet Studies for the Social Sciences (LISS) panel

**DOI:** 10.1007/s00038-016-0880-2

**Published:** 2016-08-30

**Authors:** Audrey M. W. Simons, Annemarie Koster, Daniëlle A. I. Groffen, Hans Bosma

**Affiliations:** Department of Social Medicine, CAPHRI School for Public Health and Primary Care, Maastricht University, P.O. Box 616, 6200 MD Maastricht, The Netherlands

**Keywords:** The Netherlands, Perceived classism, Meritocratic ideology, Socioeconomic status, Self-rated health, Inferiority

## Abstract

**Objectives:**

Classism might be the downside of the prevailing ideologies of individual responsibility for success. However, since studies into perceived classism have mainly been qualitative, little is known about its association with socioeconomic status, health, health behaviours and perceived inferiority, especially in more egalitarian countries. This study, therefore, examined the associations of perceived classism with socioeconomic status, health, health behaviours and perceived inferiority.

**Methods:**

We used cross-sectional data (2012/2013) from the Dutch Longitudinal Internet Studies for the Social Sciences (LISS) (*n* = 1540; age 16–90; 46.9 % men).

**Results:**

We found that classism was perceived by 18.2 % of the participants, with the lowest income and occupation group most likely to perceive classism (22.0 and 27.5 %, respectively). Perceived classism was significantly associated with poor health (e.g. self-rated health OR = 2.44, 95 % CI = 1.76–3.38) and feelings of inferiority (e.g. shame OR = 4.64, 95 % CI = 3.08–6.98). No significant associations were found with health behaviours.

**Conclusions:**

To further examine the role of perceived classism for socioeconomic differences in health and its association with country-level socioeconomic inequalities, prevailing ideologies, and objective opportunities for social mobility, we recommend more longitudinal and international studies with comparable measures of perceived classism.

## Introduction

Western societies are increasingly permeated by both the meritocratic ideology (e.g. the ‘American Dream’) and the ideology of individual responsibility. According to this ideology, economic success is based upon individual merits, such as having the right talents and working hard (De Botton 2004). Simultaneously, many people think that modern life has yielded increased opportunities for upward mobility for children from lower socioeconomic backgrounds (Kraus and Tan 2015). Whether these ideologies are based on facts or myths is debatable, but, as a downside, these ideologies might induce people to negatively judge and stereotype those ending up at the bottom of the socioeconomic hierarchy (Dahl et al. 2006).

Classism refers to the marginalisation (i.e. labelling, prejudice, discrimination, and stigmatisation) of those who are perceived to be in a lower social class (Liu 2013). People’s experience of such classism (perceived classism) has been mainly investigated in qualitative studies. These studies found that people in poverty or low socioeconomic status groups feel negatively judged, degraded, isolated, devalued, put down, blamed and looked down on by others (Collins 2005; Hirschl et al. 2011; McIntyre et al. 2003; Ravensbergen and VanderPlaat 2010; Underlid 2007). Quantitative studies have been scarce and most studies into perceived classism have been conducted in the Anglo-Saxon context (e.g. Langhout et al. 2007). Little is known about whether classism is also experienced in countries that are more egalitarian and perhaps less pervaded by the above ideologies, and whether perceived classism, just like other forms of stigmatisation and discrimination (e.g. racism) (Krieger et al. 1993; Pascoe and Smart Richman 2009), is also associated with poorer physical and mental health (Caputo 2003; Fuller-Rowell et al. 2012; Mickelson and Williams 2008; Roy 2004; Simons et al. 2013), perceptions of inferiority (Adler 2009; Courtwright 2009; Mickelson and Williams 2008; Ritsher et al. 2003; Roy 2004; Twenge and Campbell 2002; Walker et al. 2013; Williams 1999) and unfavourable health behaviours (Roy 2004).

Using cross-sectional data on more than 1500 Dutch men and women, we assessed how, in the more egalitarian Dutch context (OECD 2011), perceived classism relates to socioeconomic status (SES), health outcomes, perceptions of inferiority and health behaviours.

## Methods

### Study population

Data were collected from individuals participating in the Longitudinal Internet Studies for the Social sciences (LISS) panel (CentERdata, Tilburg, The Netherlands). This is a representative sample of the Dutch population (aged 16 years and older) who participate in monthly Internet surveys. Every year, a longitudinal survey is fielded among a panel, covering a large variety of domains including work, education, income, housing, time use, political views, values and personality (CentERdata 2011). More information about the LISS panel can be found at http://www.lissdata.nl. Ethical approval was not necessary for this study. LISS panel members have given informed consent to participate in monthly questionnaires.

In February 2013, 2656 randomly selected panel members, including multiple members of the same household, were invited to participate in a ‘perceived classism’ survey. Non-responders received two reminders for the questionnaire. The questionnaire was completed by 2096 participants (78.9 %). After excluding participants because of missing data on SES, outcome variables, or covariates measured between November 2012 and May 2013, the final sample consisted of 1540 participants (73.5 %). The mean age of the sample was 53.5 years (SD = 15.6, range 16–90), 722 participants (46.9 %) were men and 172 participants (11.2 %) were of non-Dutch origin. The design of the current study was cross-sectional.

### Measures

#### Perceived classism

Perceived classism was measured with eight statements about perceived class-related stigmatisation within the last 6 months: (1) I feel that I am odd or abnormal because of my financial situation, educational level or occupation; (2) there have been times when I have felt ashamed because of my …; (3) I never feel self-conscious when I am in public (R); (4) I never feel embarrassed about my … (R); (5) I feel that others look down on me because of my …; (6) people treat me differently because of my …; (7) I have found that people say negative or unkind things about me behind my back because of my … and (8) I have been excluded from work, school and/or family functions because of my …. Participants reported the extent to which they agreed or disagreed with each statement on a five-point Likert scale (1 = definitely disagree to 5 = definitely agree) (Mickelson and Williams 2008). The questionnaire was translated into Dutch and slightly adjusted to cover a broader definition of socioeconomic grouping. On the basis of factor and reliability analyses, we excluded two items from the original scale (items 3 and 4; these were the only negatively worded items that loaded onto a different factor). The Cronbach’s alpha of the six-item scale is 0.83. Scores were dichotomised by categorising those scoring 4 or 5 on at least one of the six items as ‘perceiving classism’ (*n* = 280, 18.2 %).

#### Socioeconomic status

Participants’ equivalent household income was defined as the net monthly household income in Euros corrected for the number of adults and children living in the household (Vrooman et al. 2007). Equivalent household income was categorised, based on tertiles, into high (>€2000, reference category), moderate (€1426–€2000) and low income (<€1425). Education was divided into three categories: (1) higher vocational education and university (reference category; *n* = 523, 34 %), (2) higher secondary education and intermediate vocational education (*n* = 529, 34.4 %) and (3) primary school and intermediate secondary education (*n* = 488, 31.7 %). Occupational level was also divided into three categories: (1) higher academic or independent profession, and higher supervisory profession (reference category; *n* = 236, 15.3 %), (2) intermediate academic or independent profession, and intermediate supervisory or commercial profession and other mental work, and skilled and supervisory manual work (*n* = 1064, 69.1 %) and (3) semi-skilled manual work, and unskilled and untrained manual work (*n* = 240, 15.6 %).

#### Health outcomes

Self-rated health was measured with the question ‘How would you describe your health, generally speaking?’ (scores ranging from 1 = poor to 5 = excellent) and dichotomised by categorising poor and moderate health as ‘less than good health’. Perceived difficulties because of health problems were measured with three questions asking the participants to what extent their physical or emotional problems had impeded their daily activities, social activities and work (e.g. in their job, housekeeping or at school) over the past month. (scores ranging from 1 = very much to 5 = not at all, Cronbach’s alpha = 0.89). These variables of perceived difficulties were averaged. Scores were dichotomised by categorising those scoring 3 or lower as ‘impeded by health’. Participants were also asked to indicate whether their health was better or worse than last year (scores ranging from 1 = considerably poorer to 5 = considerably better). Scores were dichotomised by categorising those scoring less than 3 as ‘worse subjective health’. Perceived negative emotions were measured with five items asking how often participants felt anxious, down, depressed, calm (reversed) and happy (reversed) (scores ranging from 1 = never to 6 = continuously, Cronbach’s alpha = 0.86). The five items were averaged and dichotomised by categorising those scoring 4 or higher as ‘perceiving negative emotions’.

#### Perceived inferiority

Generalised shame was measured by the three-item subscale of the Differential Emotions Scale (DES) (Izard et al. 1993) (Cronbach’s alpha = 0.83). One of the items was: ‘In your daily life, how often do you feel embarrassed when somebody sees you make a mistake’ (scores ranging from 1 = never to 5 = very often). Scores were dichotomised by categorising those scoring 4 or 5 on one of the items as ‘perceiving shame’. Social anxiety was measured by 15 items of the Social Inadequacy subscale of the Dutch Personality Questionnaire (Luteijn et al. 1985) (Cronbach’s alpha = 0.87). One of the items was: ‘I get nervous when I’m going to meet people’ (true/not true/?). Scores were dichotomised using gender-specific norms for sum scores (Luteijn et al. 1985); scores above the norm were categorised as ‘perceiving social anxiety’. Self-esteem was measured with Rosenberg’s ten-item Self-Esteem Questionnaire (*n* = 1397; Cronbach’s alpha = 0.90). Participants reported the extent to which they agreed or disagreed with ten statements with seven-point Likert scales (scores ranging from 1 = totally disagree to 7 = totally agree). One of the items was: ‘I feel I do not have much to be proud of’ (reversed). Scores were dichotomised by categorising those with sum scores less than 53 as ‘low self-esteem’ (lowest tertile).

#### Health behaviours

Alcohol use was measured with the question ‘On how many of the past 7 days did you have a drink containing alcohol?’ More than two times a week was categorised as ‘alcohol use >2 days a week’. Participants who reported to be current smokers were categorised as ‘current smokers’. Physical activity was measured by two questions ‘Looking back on the last 7 days, on how many of those days did you perform a strenuous physical activity such as lifting heavy loads, digging, aerobics or cycling?’ and ‘Looking back on the last 7 days, on how many of those days did you perform a moderately intensive physical activity such as carrying light loads, cycling at a normal pace or a doubles game of tennis?’. Participants scoring zero (=no physical activity in the past 7 days) on both items were categorised as ‘inactive’. Obesity was defined as a body mass index (BMI) ≥30, calculated as self-reported weight (kg) divided by height (m) squared.

#### Covariates

Covariates were age (years), sex and ethnicity [Dutch (=reference category) or first or second-generation immigrant with Western or non-Western background].

### Statistical analyses

First, associations between socioeconomic status (income, education and occupation) and perceived classism were examined by *χ* ^2^ tests and by logistic regression analyses adjusted for the covariates age, sex and ethnicity. Second, the effect of perceived classism on health outcomes, perceived inferiority and health behaviours was examined using logistic regression analyses with additional adjustments for age, sex, ethnicity (model 2) and income (model 3). Finally, sensitivity analyses were performed (e.g. adjustments for education and occupation instead of income, an assessment of dose–response associations of perceived classism). All analyses were performed with IBM SPSS Statistics for Windows, Version 22.0 (IBM corp., Armonk, NY, USA).

## Results

Almost one-fifth of the participants perceived some form of classism [280 (18.2 %) out of 1540 people; Table 1]. The two groups differed in age and sex; the group perceiving classism was, on average, younger (49.5 vs. 54.4 years) and included more men (53.6 vs. 45.4 %). Participants who perceived classism were also more likely to report health problems and feelings of inferiority than their counterparts (e.g. 26.4 vs. 15 % reporting less than good health, and 23.6 vs. 14.3 % reporting social anxiety). As regards health behaviours, the groups only differed significantly in smoking behaviour, as 25.7 % of the participants who perceived classism smoked, compared to 19.0 % of the participants who perceived no classism.

**Table 1:** Baseline characteristics. Source: Longitudinal Internet Studies for the Social Sciences panel, The Netherlands, 2013

	Total (*n* = 1540)	Perceived classism (*n* = 280)	No perceived classism (*n* = 1260)
Age, mean (SD)	53.47 (15.56)	49.46 (15.47)	54.36 (15.45)**
Men	46.9	53.6	45.4*
Non-Dutch	11.2	14.3	10.5
Income^a^			
High	33.2	24.3	35.2*
Moderate	33.1	35.0	32.7
Low	33.7	40.7	32.1
Occupational level			
High	15.3	16.1	15.2**
Moderate	69.1	60.4	71.0
Low	15.6	23.6	13.8
Educational level			
High	34.0	36.4	33.4
Moderate	34.4	33.9	34.4
Low	31.7	29.6	32.1
Less than good health	17.1	26.4	15.0**
Impeded by health	14.4	23.9	12.2**
Worse subjective health	18.6	23.6	17.5*
Negative emotions	4.8	10.4	3.6**
Shame	8.1	20.4	5.3**
Social anxiety	16.0	23.6	14.3**
Low self-esteem^a^	30.8	40.9	28.7**
Currently smoking	20.2	25.7	19.0*
Alcohol use >2 days a week	38.1	35.7	38.7
Inactivity	25.0	24.3	25.2
BMI > 30	15.1	16.8	14.7

* *p* value χ^2^ < 0.05, ** *p* value χ^2^ < 0.001

^a^Tertiles

^b^Agricultural professions (*n* = 26) excluded, because it was unclear whether they were farm workers (i.e. low status) or independent farmers (i.e. high status)

Respondents in the lowest income and occupation group were significantly more likely to experience classism than those with the highest SES (22.0 vs. 13.3 %, and 27.5 vs. 19.1 %, respectively) (Table 2). Adjusted for age, sex and ethnicity, the lowest income and occupation group had 1.88 (95 % CI = 1.34–2.63) and 1.57 (95 % CI = 1.00–2.46) times higher odds of perceiving classism than their better-off counterparts. Associations with education were not statistically significant. A gradient-like association was found for income.

**Table 2:** Association between socioeconomic status and perceived classism, *n* = 1540. Source: Longitudinal Internet Studies for the Social Sciences panel, The Netherlands, 2013

	Perceived classism				
	Model 1			Model 2	
	%^a^	OR	95 % CI	OR	95 % CI
Total	18.2				
Income					
High	13.3	Ref		Ref	
Moderate	19.2	1.55	(1.11–2.17)	1.53	(1.09–2.15)
Low	22.0	1.83	(1.32–2.55)	1.88	(1.34–2.63)
Occupational level					
High	19.1	Ref		Ref	
Moderate	15.9	0.80	(0.56–1.15)	0.87	(0.59–1.26)
Low	27.5	1.61	(1.05–2.48)	1.57	(1.00–2.46)
Educational level					
High	19.5	Ref		Ref	
Moderate	18.0	0.90	(0.66–1.23)	0.87	(0.64–1.19)
Low	17.0	1.01	(0.73–1.40)	1.01	(0.73–1.40)

Model 1 = unadjusted OR, model 2 = model 1 + adjustment for age, sex and ethnicity

^a^Percentage agreed with at least one of the items

Table 3 shows that, adjusted for age, sex, ethnicity and income (model 3), people who perceived classism had 2.44 times higher odds (95 % CI = 1.76–3.38) of reporting less than good health, 2.43 times higher odds (95 % CI = 1.74–3.41) of feeling impeded by health problems, 1.71 times higher odds (95 % CI = 1.24–2.36) of reporting worse health compared to a year ago, and 2.97 times higher odds (95 % CI = 1.80–4.90) of reporting negative emotions, compared to those who perceived no classism.

**Table 3:** Association between perceived classism and health outcomes, *n* = 1540. Source: Longitudinal Internet Studies for the Social Sciences panel, The Netherlands, 2013

	Health							
	Less than good health		Impeded by health		Worse subjective health		Perceived negative emotions	
	OR	(95 % CI)	OR	(95 % CI)	OR	(95 % CI)	OR	(95 % CI)
Model 1	2.04	(1.50–2.77)	2.26	(1.64–3.12)	1.45	(1.06–1.98)	3.12	1.92–5.07
Model 2	2.53	(1.83–3.50)	2.59	(1.86–3.62)	1.66	(1.21–2.29)	3.22	1.96–5.29
Model 3	2.44	(1.76–3.38)	2.43	(1.74–3.41)	1.71	(1.24–2.36)	2.97	1.80–4.90

Model 1 = unadjusted OR, model 2 = model 1 + adjustment for age, sex and ethnicity, model 3 = model 2 + adjustment for income

Similarly, Table 4 shows that, adjusted for age, sex, ethnicity and income (model 3), people who perceived classism also had significantly higher odds of reporting feelings of shame (OR = 4.64, 95 % CI = 3.08–6.98), social anxiety (OR = 1.69, 95 % CI = 1.22–2.34) and low self-esteem (OR = 1.65, 95 % CI = 1.23–2.22) than those who did not report perceiving classism.

**Table 4:** Association between perceived classism and perceived inferiority, *n* = 1540. Source: Longitudinal Internet Studies for the Social Sciences panel, The Netherlands, 2013

	Perceived inferiority					
	Perceived shame		Perceived social anxiety		Low self-esteem	
	OR	(95 % CI)	OR	(95 % CI)	OR	(95 % CI)
Model 1	4.55	3.11–6.66	1.85	1.35–2.54	1.72	1.29–2.29
Model 2	4.68	3.12–7.00	1.72	1.28–2.45	1.75	1.31–2.35
Model 3	4.64	3.08–6.98	1.69	1.22–2.34	1.65	1.23–2.22

Model 1 = unadjusted OR, model 2 = model 1 + adjustment for age, sex and ethnicity, model 3 = model 2 + adjustment for income

Self-esteem *n* = 1397

The association between perceived classism and smoking was significant in the unadjusted model and in the model adjusted for age, sex and ethnicity (OR_unadjusted_ = 1.48, 95 % CI = 1.09–2.00), but lost its significance after adjusting for income. Associations between perceived classism and alcohol use, inactivity and BMI were not statistically significant (Table 5, model 3).

**Table 5:** Association between perceived classism and health behaviours, *n* = 1540 Source: Longitudinal Internet Studies for the Social Sciences panel, The Netherlands, 2013

	Health behaviours							
	Alcohol use >2 days a week		Current smokers		Inactive		BMI ≥ 30	
	OR	(95 % CI)	OR	(95 % CI)	OR	(95 % CI)	OR	(95 % CI)
Model 1	0.88	0.67–1.16	1.48	1.09–2.00	0.95	0.71–1.29	1.17	0.83–1.66
Model 2	0.99	0.74–1.31	1.40	1.03–1.90	1.02	0.75–1.39	1.25	0.88–1.79
Model 3	1.05	0.78–1.40	1.35	0.99–1.84	0.99	0.73–1.36	1.21	0.85–1.74

Model 1 = unadjusted OR, model 2 = model 1 + adjustment for age, sex and ethnicity, model 3 = model 2 + adjustment for income

In additional analyses, we first examined the interactions between perceived classism and age, sex, ethnicity and SES; these were not statistically significant (all *p* > 0.10). Second, we alternatively adjusted for occupation and education (instead of income) in the associations between perceived classism and the outcomes (not tabulated). These analyses did not yield different results, although the association between perceived classism and smoking remained statistically significant after adjusting for education (OR = 1.43, 95 % CI = 1.05–1.95). Third, dose–response associations were found between perceived classism (categorised into three groups based on sum score tertiles) and health outcomes and perceived inferiority measures (not tabulated). Finally, analyses with only one randomly selected participant per household (*n* = 1352) confirmed the pattern of findings presented above.

## Discussion

Almost one in five of the participants of a Dutch internet panel perceived some kind of classism, with the lowest income and occupation group most likely to report it (22.0 and 27.5 %, respectively). Low education was not associated with perceived classism. Perceptions of classism were strongly associated with poor physical and mental health and perceptions of inferiority. Perceived classism was not associated with unhealthy behaviours.

Our results might provide support for the relevance of the relative deprivation theory in social epidemiology (Wilkinson and Pickett 2007). Inequalities in society, whether large or small, together with prevailing ideologies of social mobility and individual responsibility, might nourish feelings of inferiority and perceptions of classism in lower SES groups (De Botton 2004; Roy 2004). The concept of relative deprivation might also explain why we found no differences in perceived classism between groups based on educational level. When comparing one’s status with that of relevant others, one might typically look at the more visible indicators of status, like someone’s possessions (e.g. expensive cars as an indicator of someone’s wealth) or occupation (De Botton 2004). More visible indicators of status, or in this case the visible lack of it, might be more prone to stigmatisation, and these might be more difficult to conceal to others than a low level of education (Quinn 2006).

In view of its relation with poor physical and mental health, and its highest prevalence in the lowest SES groups, perceived classism might even be a relevant but largely neglected factor in social epidemiology and particularly in research to explain the persistent socioeconomic health inequalities (Fuller-Rowell et al. 2012; Hatzenbuehler et al. 2013; Krieger 2014; Simons et al. 2013). Moreover, if classism, as an antecedent of perceived classism, is a relevant factor, it will be very difficult to achieve its prevention by public health programmes aimed at tackling socioeconomic inequalities in health, as classism is embedded in hard-to-change, ingrained ideologies. A more practical approach might be to create opportunities for individuals to cope differently with the experience of classism. Our next, as yet unpublished, qualitative work will highlight the most important healthy and unhealthy ways of coping with classism. Intervention measures may take these into account in trying to reduce health inequalities. Most importantly, however, social epidemiology needs more longitudinal research on how exactly classism (enacted or perceived) is linked to socioeconomic inequalities in health and how much it contributes to these inequalities.

There is a lack of comparable international data on classism; most data stem from qualitative studies or from studies among specific populations (e.g. students; Langhout et al. 2007). We therefore, cannot conclude that people in a rather egalitarian country, such as The Netherlands (OECD 2011), perceive more or less classism than people in, for example, the US. More research is needed to study the prevalence of classism in different parts of the world, using the same measurement instruments. In addition, by measuring perceived classism worldwide, it would for example be possible to assess how the prevalence of perceived classism and its relation to health vary between countries differing in terms of socioeconomic inequality (e.g. income inequality), ideologies (e.g. meritocratic vs. egalitarian) and opportunities for social mobility (Bosma et al. 2012; Bullock 2006; Dahl et al. 2006; Rüsch et al. 2010; Simons et al. 2013; Swierstra and Tonkens 2011; Underlid 2005; Williams 2009).

Moreover, if the role of classism and its experience in individual and population health is further substantiated, time trends in classism within countries also become relevant. If perceived classism were measured regularly, unintended effects of changing national policies could become clear. For example, the Dutch welfare state is changing into what politicians call a ‘participation society’, which emphasises taking individual responsibility. This might increase negative attitudes towards people who are not able to participate (Hindriks 2015), resulting in more people perceiving stigmatisation, because of their lack of participation, education, income or employment.

Although our study confirmed the association between perceived classism, health and perceived inferiority, we could not confirm the association between perceived classism and unhealthy behaviours. This might indicate that unhealthy behaviours are not included in the pathways relating perceived classism to poor health. An alternative pathway by which perceived classism affects health might involve physiological stress responses, related to neurochemical, endocrine and immunological functioning, which are associated with both physical and mental health problems (Baum et al. 1999; Courtwright 2009; Hatzenbuehler et al. 2013; Krieger 2014; Roy 2004). This also needs further examination in future studies.

The major strength of our study was the use of a large representative Dutch Internet panel (De Vos 2010). Where necessary, computers and an Internet connection were provided to people—most often people from the low SES groups, elderly people or people with non-Dutch ethnic backgrounds. Nevertheless, some methodological issues have to be discussed. First, as we used cross-sectional data, we cannot draw any causal conclusions. Poor health and feelings of inferiority could be causes of perceived classism, rather than consequences. Similarly, there might be personality characteristics, e.g. relating to negative affectivity, that are confounders of the cross-sectional associations, particularly as measures were based on self-reports. Hence our recommendation to corroborate our hypotheses in longitudinal research, which would enable us to more validly study the relevant causal processes. Second, the non-responders in our study (21 %) differed significantly from the respondents in terms of age (*M* = 39.20, SD = 16.17 vs. *M* = 50.79, SD = 17.24), and participants who were excluded from the analyses because of missing data were also significantly younger (*M* = 43.40, SD = 19.37 vs. *M* = 53.47, SD = 15.56), and were more likely to perceive classism (24.3 vs. 18.2 %) and to belong to the lower income and education group (49.5 vs. 33.7 %, and 40.3 vs. 31.7 %, respectively). This pattern may have resulted in underestimated associations.

### Conclusion

Despite living in a rich and relatively egalitarian country, almost 20 % of the participants in a Dutch Internet panel perceived some kind of classism. The lowest income and occupation groups were most likely to perceive classism. Comparable international data are needed to assess between-country differences in perceived classism and the role of country characteristics, like the prevailing ideologies or the opportunities for social mobility. Furthermore, because of the strong associations we found between SES, perceived classism, poor health and perceptions of inferiority, future longitudinal research should shed further light on the role of perceived classism in social epidemiology.
